# Supplementary figures and images for: Capsid structure of a fungal dsRNA megabirnavirus reveals its previously unidentified surface architecture
Source: PLoS Pathog. 2023 Feb 27;19(2):e1011162. doi: 10.1371/journal.ppat.1011162 (PMC9997902; doi:10.1371/journal.ppat.1011162)

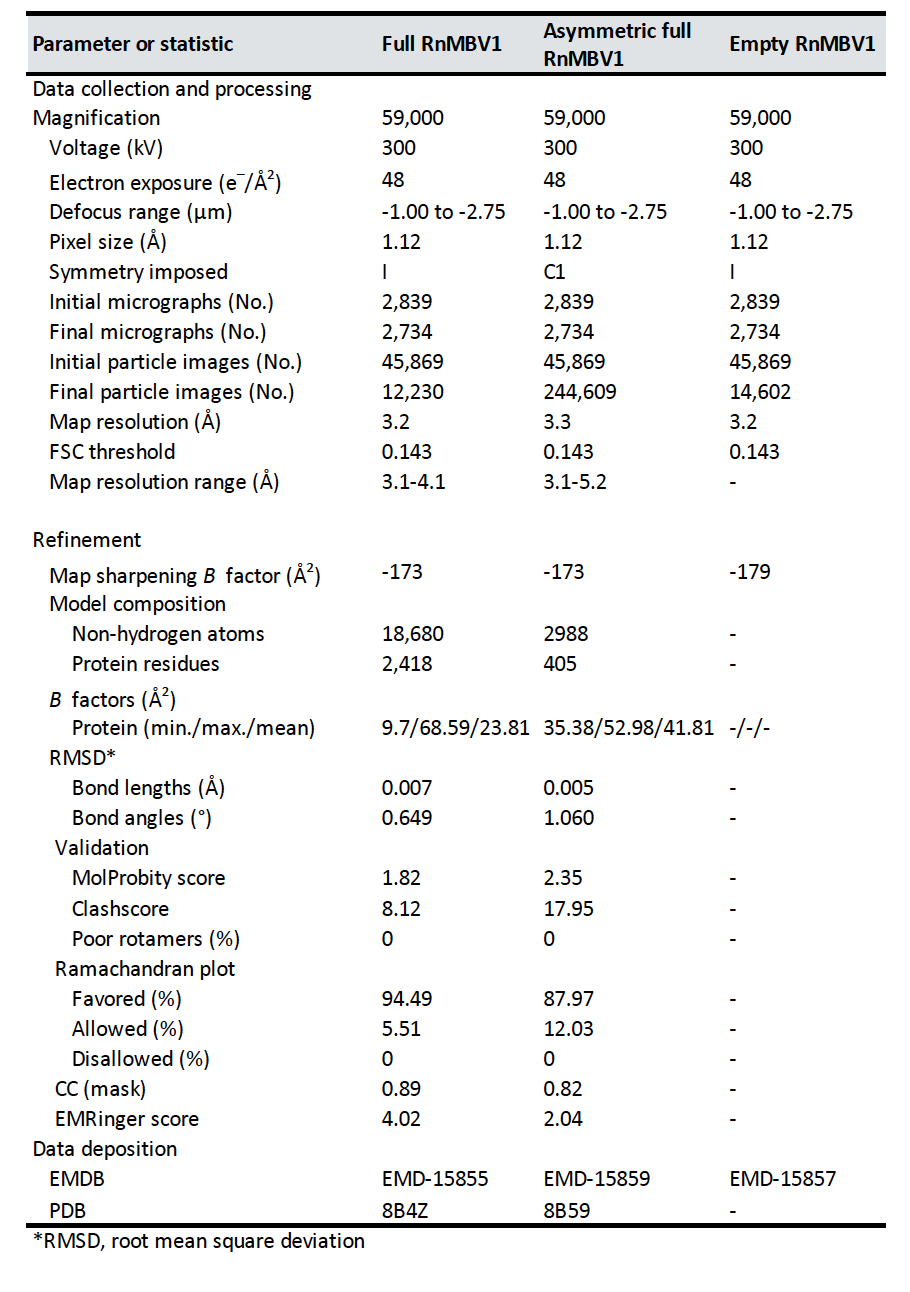

Supplement: S1 Table — (PNG) [file ppat.1011162.s001.png]

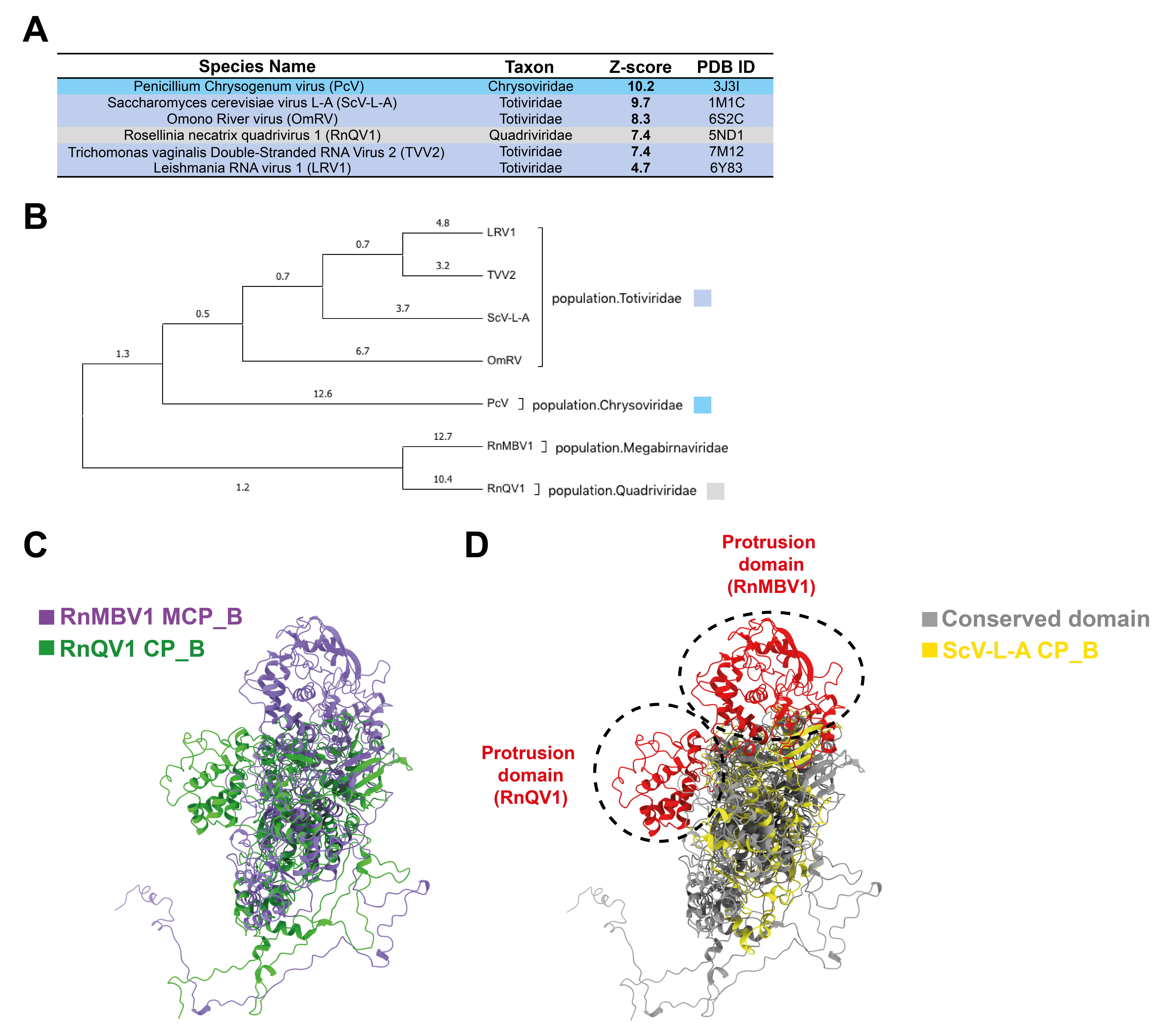

Supplement: S2 Fig — A) Summary table of viral CP structures similar to that of the RnMBV1 MCP identified using Dali search. The background colors indicate different taxonomy clades. B) Structure-based phylogenetic tree of RnMBV1 MCP and structurally close-related six CPs. C) Superimposition of RnMBV1 MCP and RnQV1 CP. D) Superimposition of RnMBV1 MCP, RnQV1 CP, and yeast ScV-L-A CP. Dashed circles highlight protrusion domains of RnMBV1 and RnQV1. (PNG) [file ppat.1011162.s003.png]

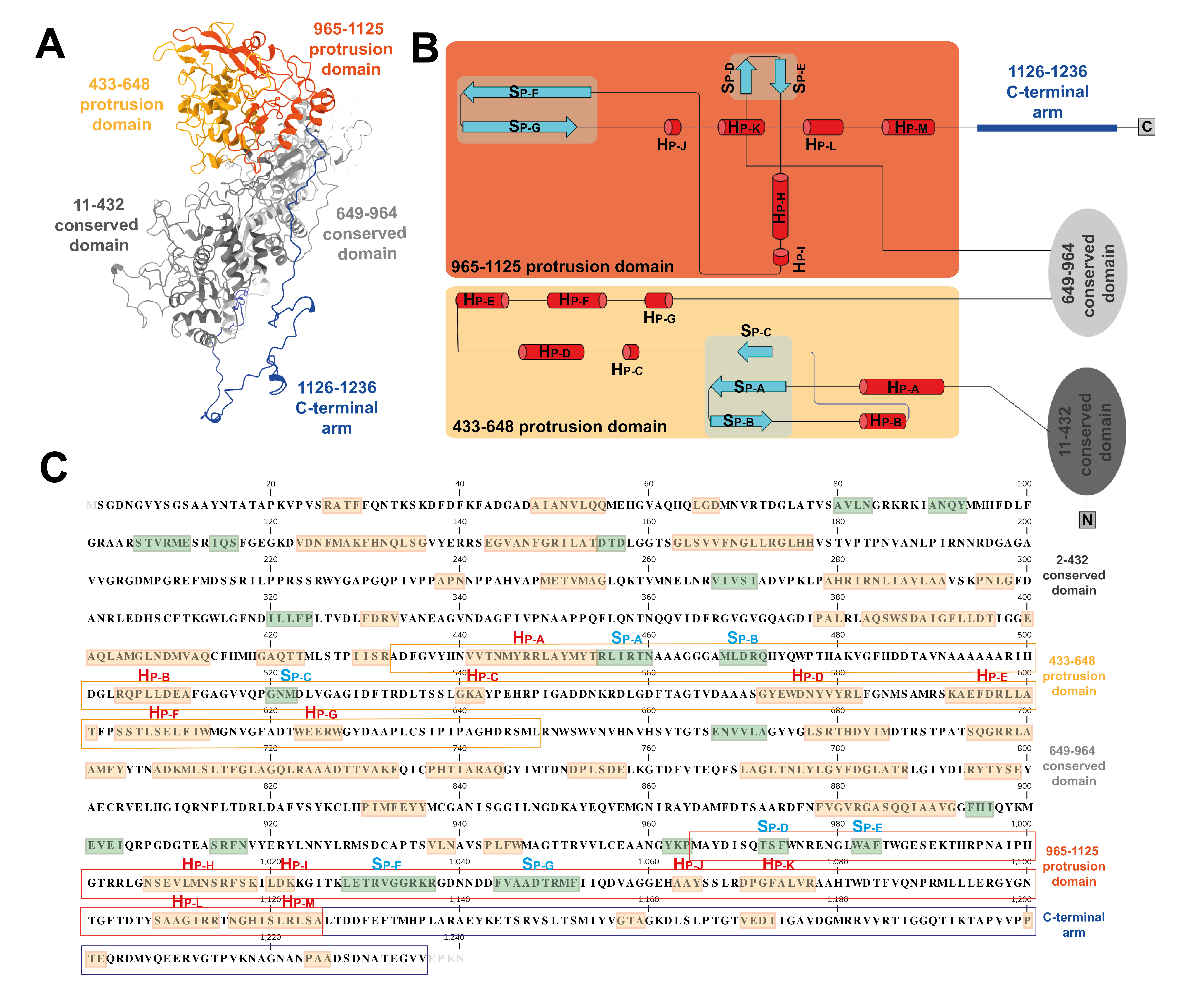

Supplement: S3 Fig — A) Atomic model of one MCP colored by domains. The protrusion domains (amino acid residues 433–648 and 965–1125) are colored yellow and orange, respectively and the conserved domains (amino acid residues 11–432 and 649–964) are colored dark gray and light gray. The C-terminal arm (amino acid residues 1126–1236) is colored dark blue. B) Structural topology diagram of the MCP. The color codes correspond to those in A). The ɑ-helices and β-strands are shown as red cylinders and light blue arrows and named accordingly. The blue, translucent, rounded boxes cover the regions containing β-sheets. C) Amino acid sequence organization of the MCP. The orange and green boxes highlight ɑ-helices and β-strands, respectively. The colors and labels correspond to those of B). (PNG) [file ppat.1011162.s004.png]
