# Supplementary material for: Capsid structure of a fungal dsRNA megabirnavirus reveals its previously unidentified surface architecture
Source: PLoS Pathog. 2023 Feb 27;19(2):e1011162. doi: 10.1371/journal.ppat.1011162 (PMC9997902; doi:10.1371/journal.ppat.1011162)
Supplement: Attachment — Submitted filename: Reponse_to_reviewers.docx [file ppat.1011162.s005.docx]

**Point-to-point response to reviewers**

***Part I - Summary***

***Reviewer #1:*** *This manuscript presents a high-resolution structure of a megabirnavirus building up on an earlier low resolution structure. It identifies molecular structures that are proposed to be unique to this virus. This structural study describes the first molecular structure for this viral family and will be of interest to the field.*

*However, the structure description and figures need to be improved as described in Part III of the review.*

**Response**: We appreciate your comments so much. We have carefully improved the figures and main text suggested in Part III of your review.

***Reviewer #2:*** *The manuscript by Wang et al. describes the cryoEM-based atomic model (3.2-Å resolution) of Rosellinia necatrix megabirnavirus 1-W779, which is a nonenveloped icosahedral dsRNA virus that infects the ascomycete fungus Rosellinia necatrix, a causative agent of the lethal plant disease white root rot.*

*Basic features of the capsid structure formed by the dsRNA1-encoded major capsid protein (MCP) are not unexpected for a dsRNA virus with a 120-subunit T=1 (so-called "T=2") protein shell. There is an especially long C-terminal extension that makes multiple contacts across different subunits, but such terminal extensions, though often not as long, have been seen in other "T=2" capsids. The protrusion domain, whose function remains unknown, appears unique, but having a unique (e.g., species- or genus-specific) protrusion domain appears to be the rule rather than the exception among dsRNA viruses with "T=2" capsids.*

*Probably the most interesting observation is the presence of an unexpected protein trimer anchored atop the 3-fold axes and attributable to the dsRNA3-encoded protein, about which little was previously known but which is now designated the crown protein (CrP). The CrP trimer is apparently not present at every 3-fold axis based on density/occupancy analyses. The authors argue for a role of this protein in horizontal transmission of this virus.*

*Given interesting aspects of the CrP, I suggest the following additions to the text:*

*a. The reduced occupancy of the CrP is given very little attention in the text, in fact a single sentence in Results and Discussion: "The CrPs are not present at all 3-fold axes (Figs. S2 and S3), which is probably due to their limited and weak interactions with MCPs (Fig. 4B)." I would like to see this given a bit more emphasis, perhaps by including one of the supplementary figures in the main article and summarizing the nature of the analyses in Results and Discussion. I also think the authors should acknowledge the somewhat trivial explanation that some of the CrP trimers might have been lost during purification.*

**Response**: Thank you for these constructive comments. We have moved Supplementary Figure S2 and S3 to the main text, and now they are shown as Fig. 6 and 7. As you pointed out, the limited and weak interactions of the CrPs are intriguing to discuss with the results in new Figs. 6 and 7. We emphasized this point and include the possibility of losing the CrPs during purification steps (Page 11, Lines 235-237).

*b. The authors state "The weak binding between the CrPs trimer and MCPs, however, indicates that the CrPs do not function crucially in the [virus-to-host] transmissions." I don't consider this a logically necessary or even probable conclusion. Full occupancy of this protein would not necessarily be required for playing a crucial role in whatever its function might be.*

**Response**: We have reconsidered this point according to your comment and our results. As commented, it is very likely that the full occupancy of the CrPs is not necessary for their function. We thus modified this sentence considering your advice (Page 12, Lines 244-247).

*c. The authors state "Fungal viruses are horizontally transferred to R. necatrix from unidentified organisms in the soil (49,50), implying the presence of vector organisms. It is of interest to speculate that the CrPs are associated with horizontal virus transmission under natural conditions." I agree that this seems like an interesting possibility worth discussing, but I feel this idea comes out of the blue. There is no preceding mention of vectors in this manuscript. I think the authors should explain this concept a bit more clearly and how the observations in refs. 49 and 50 might point to it.*

**Response**: We have taken your comment in consideration, and a few preceding statements about mycovirus horizontal transmission have been added with additional references newly cited for the discussion (Page 12, Lines 253-261).

*Final comment, Title: I think the title could be improved. The phrase "uniquely acquired structures" doesn't tell us much, and "acquired" from where? Also, is the term "metazoan" really needed?*

**Response**: We agree that the previous title is not addressing critical points of our study. We removed “uniquely acquired structures” and “metazoan”, and instead mentioned “previously unidentified surface architecture” in our new title.

***Part II – Major Issues: Key Experiments Required for Acceptance***

***Reviewer #1:*** *Validation reports need to be submitted as obtained from the EMDB and the PDB.*

*CC mask is 0 for the C1 reconstruction…*

**Response**: The validation reports are provided additionally (EMD-15855_8B4Z_validation_reports.pdf; EMD-15857_validation_reports.pdf; EMD-15859_8B59_validation_reports.pdf). Thank you so much for your careful check. The incorrect CC (mask) value for the C1 reconstruction was written in the previous version. We ran validation job again and reconfirmed that the CC (mask) value is now 0.82. We also updated some statistics for C1 reconstruction, shown in Table. S1.

***Reviewer #2:*** *None to note*

***Part III – Minor Issues: Editorial and Data Presentation Modifications***

***Reviewer #1:*** *It is important that the fold of the MCP and CrP are introduced in the first couple of figures. A cartoon representation and box diagram of the domains is essential for readability (e.g. bring Fig. S6 into the main text).*

**Response**: Fig. S6 has been moved into main text and it is now Fig. 4.

*In addition, the role of the C-terminal arm is interesting, but it is hard to follow the interaction network on individual subunits. Could this be represented over half a capsid? The labels are too small and unreadable even when zooming in.*

**Response**: We have improved the labels in Fig. 2 as much as we can. To address your point, a panel D was added to Fig. 2. Panel D represents the entire interaction network in 5-fold complex (ten MCP subunits) of the capsid with all C-terminal arms highlighted. We hope that this panel makes it easier to follow the interaction network.

*Fig S4 is also impossible to follow for panels B, D, E because too many structures are superimposed. Consider using a single example or a stereo-image.*

**Response**: We have rearranged Fig. S4 (now renumbered as Fig. S2). We deleted panel B to avoid complexity. We recolored the atomic models and only showed a single example to make them clear in new panels.

*Title: What is a “metazoan” fungal virus? P.12, line 212 suggests that RnMBV1 is a metazoan virus, which is confusing.*

**Response**: We have meant that “metazoan fungal virus” is the virus that infects multicellular fungus. However, it is a confusing term, thus we modified this sentence to make it clearer (Page 10, Line 214; Page 11, Line 238).

*Abstract: speculations about genome packaging are not supported or discussed in the main text and should be removed.*

**Response**: We removed the term “genome packaging” in Abstract since it is still very speculative and not supported.

*Detailed comments: text editing recommended. Frequent typos (e.g. p. 10 Megabirnabiridae).*

**Response**: Thank you. We corrected this typo (Page 10, Line 216).

*p.5, l.81: clarify why the protruding proteins are “unexpected”.*

**Response**: According to your comment, we now think “previously unrecognized” is more suitable. We have changed it (Page 5, Lines 80-81).

*p.8: what does the RMSD represent (all atoms, main chain…)?*

**Response**: The RMSD here represents for all atoms. We have added the explanation in main text (Page 8, Line 150).

*p.8, l. 164: Is this “deletion” a removal of C-terminus in silico?*

**Response**: Yes, we did it in silico using software Chimera. We added this information (Page 8, Line 169).

*p.9 l.178: edit “dimerically”*

**Response**: We changed it to dimer and rewrote the whole sentence to make it clear (Page 9, Lines 178-179).

*p.9 l.190: Is the C-terminus really expected to play a role in replication during assembly as suggested here without further evidence? This needs to be clarified or edited.*

**Response**: As you pointed out, it is too speculative that the C-terminal arm is critical in viral replication. We toned down this sentence and rewrote them in Page 9, Lines 188-193. The C-terminus of a few CPs is fused with an RdRp to form a CP-RdRp complex in some non-enveloped icosahedral viruses including megabirnavirus. Namely, it is possible that the C-terminal arm could play an important role in viral encapsidation with the CPs and the CP-RdRps.

*p. 11: “Extra-surface CrPs”. Please reword.*

**Response**: We have replaced the subtitle by “Surface CrPs atop 3-fold axes”.

*p. 11, l.233: “CrP of RnMBV1 can be associated with the protrusion protein/fiber…”. I’m not clear about what’s meant here. Is CrP analogous to the fiber or physically associated? Why would it be involved in egress and/or entry for an intracellular virus?*

**Response**: Previous sentences are not clear; thus, we rewrote the sentences in Page 11, Lines 238-243. There is no sequence or structural similarity between the megabirnavirus CrP and the toti-like virus protrusion protein/fiber or the sedoreovirus outer capsid protein. We have mentioned this point clearly in Page 11, Lines 242-243. We agree that the CrPs are not important for extracellular transmission since this megabirnavirus is an intracellular virus. However, it is probably associated with horizontal transmission of the multicellular fungal megabirnavirus, as also suggested by the other reviewer who wanted us to discuss more. We clearly discussed this possibility in Page 11, Line 243 – Page 12, Line 261.

***Reviewer #2:*** *None to note except as noted in Part I.*
